# Dietary Methionine Enhances Portal Appearance of Guanidinoacetate and Synthesis of Creatine in Yucatan Miniature Piglets^☆^

**DOI:** 10.1016/j.tjnut.2024.03.017

**Published:** 2024-03-26

**Authors:** Mahesha U Asiriwardhana, Olupathage C Dinesh, Janet A Brunton, Robert F Bertolo

**Affiliations:** Department of Biochemistry, Memorial University of Newfoundland, St. John's, NL, Canada

**Keywords:** Methionine, creatine, GAA supplementation, GAA absorption, piglets

## Abstract

**Background:**

Creatine plays a significant role in energy metabolism and positively impacts anaerobic energy capacity, muscle mass, and physical performance. Endogenous creatine synthesis requires guanidinoacetic acid (GAA) and methionine. GAA can be an alternative to creatine supplements and has been tested as a beneficial feed additive in the animal industry. When pigs are fed GAA with excess methionine, creatine is synthesized without feedback regulation. In contrast, when dietary methionine is limited, creatine synthesis is limited, yet, GAA does not accumulate in plasma, urine, or liver.

**Objective:**

We hypothesized that portal GAA appearance requires adequate dietary methionine.

**Methods:**

Yucatan miniature piglets (17–21 d old; *n* = 20) were given a 4 h duodenal infusion of complete elemental diets with supplemental GAA plus 1 of 4 methionine concentrations representing either 20%, 80%, 140%, or 200% of the dietary methionine requirement. Arterial and portal blood metabolites were measured along with blood flow to determine mass balance across the gut. [^3^H-methyl] methionine was infused to measure the methionine incorporation rate into creatine.

**Results:**

GAA balance across the gut was highest in the 200% methionine group, indicating excess dietary methionine enhanced GAA absorption. Creatine synthesis in the liver and jejunum was higher with higher concentrations of methionine, emphasizing that the transmethylation of GAA to creatine depends on sufficient dietary methionine. Hepatic GAA concentration was higher in the 20% methionine group, suggesting low dietary methionine limited GAA conversion to creatine, which led to GAA accumulation in the liver.

**Conclusions:**

GAA absorption and conversion to creatine require a sufficient amount of methionine, and the supplementation strategies should accommodate this interaction.

## Introduction

Guanidinoacetic acid (GAA) is an amino acid derivative and endogenous substance in body tissues [1,2]. GAA acts as an immediate precursor for creatine, which plays a key role in energy metabolism, especially in muscle [3] and brain [4]. Because of its role in muscle energetics, creatine supplementation has been popular as a performance-enhancing agent among athletes [5] as well as in the animal production industry [6,7]. However, creatine supplementation has significant drawbacks, including instability during the manufacturing process, high cost [8], and relatively low bioavailability [9]. Hence, supplemental GAA has been proposed as a potential alternative approach to enhance creatine availability in the body. Moreover, GAA also seems to have several beneficial noncreatine roles in cellular metabolism. For example, GAA can directly or indirectly affect endocrine functions, neuromodulation, and oxidant–antioxidant processes [10,11].

Endogenous creatine synthesis and dietary creatine intake are essential to replenish daily creatine losses, estimated to be 1.7% of the body pool per day in young humans [12]. Moreover, plant-based diets may not contain sufficient amounts of creatine, which would then need to be synthesized entirely. Similarly, although neonatal piglets consume milk that is high in creatine, they still need to produce 80% of their creatine, because demands for their rapid growth exceed dietary supply [13]. Creatine biosynthesis involves 2 enzymes and 3 amino acids (i.e., arginine, glycine, and methionine) in an interorgan process. In the kidney, arginine transfers its amidino group to glycine to synthesize GAA and ornithine via arginine:glycine amidino transferase (AGAT). GAA produced by the kidney is transported to the liver, where it is methylated using a methyl group from S-adenosylmethionine (SAM), which is a direct metabolite of methionine and the primary methyl donor in the body. This reaction produces creatine and S-adenosylhomocysteine and is catalyzed by guanidinoacetate methyltransferase (GAMT) [12]. This interorgan pathway is responsible for most whole-body creatine synthesis; however, creatine biosynthesis is not restricted to these tissues and occurs at varying rates in other organs [13]. In pigs, GAMT activity is highest in the liver, although it is somewhat ubiquitous, whereas AGAT occurs mainly in the kidney. The pancreas also has considerable AGAT activity in pigs [13], but >80% of total GAA is released from the kidney, suggesting the kidney is quantitatively the most important source of GAA for whole-body creatine synthesis in piglets [14].

GAA can also be fed to provide a direct precursor for creatine synthesis. We have shown that supplemental GAA readily converts to creatine, but only when sufficient methionine is available to transmethylate GAA to creatine in the liver (via SAM) [15]. Indeed, other studies have shown that supplemental GAA with methionine has beneficial effects on animal performance [16,17]. However, the fate of unconverted GAA, when methionine is limiting, is unclear. In our recent study, when dietary methionine was limited (80% of requirement), creatine synthesis was limited, yet GAA did not appear to accumulate in plasma, intestinal mucosa, kidney, brain, muscle, or liver [18]. One possibility is that absorption of supplemental GAA across the gut depends on sufficient dietary methionine; however, the effect of dietary methionine on GAA absorption across the gut is unknown. The role of the gut in creatine synthesis has not been extensively studied, and little is known about dietary methionine requirements for the de novo creatine synthesis in the gut.

The neonatal piglet is widely regarded as the closest nonprimate model for the human infant and is an ideal animal model for studying nutritional metabolism, because it shares similarities with the infant in terms of gastrointestinal tract morphology, physiology, and metabolic changes during development [[19], [20], [21]]. Moreover, the piglet has been extensively utilized in studies examining the requirements of amino acids, and their metabolism has been shown to respond to rapid dietary modifications, including changes in the composition of amino acids in the diet [22]. Therefore, the current study utilized Yucatan miniature piglets as an experimental model to investigate amino acid metabolism. We hypothesized that dietary GAA appearance in the portal vein requires adequate dietary methionine. The main objective of this study was to determine the effects of dietary methionine on supplemental GAA absorption and creatine synthesis in neonatal piglets. Furthermore, we evaluated the distribution of GAA and creatine in Yucatan miniature pigs supplied with different methionine concentrations in the diet.

## Methods

### Reagents

[^3^H-methyl]-methionine was obtained from American Radiochemicals, Inc. All other chemicals were of analytical grade and were from Sigma or Fisher Scientific.

### Piglets and surgical procedures

All animal protocols were approved by the Institutional Animal Care Committee of the Memorial University of Newfoundland in accordance with the guidelines of the Canadian Council on Animal Care. Yucatan miniature piglets (*n* = 20; 11/9 M/F, 17–21 d old, weight, 2.46–3.38 kg) were removed from the sows at the Memorial University of Newfoundland breeding colony and transported to the Animal Care Facilities on campus. Piglets were feed-restricted for 3 h before surgery to ensure that the test nutrients to be administered had no interaction with the gut contents from suckling. Piglets were randomly assigned to 1 of the 4 different infusion treatments and balanced for gender (i.e., 2–3 males per group) and weight across treatments. Piglets were sedated, intubated, and anesthetized, as described previously [23]. Under general anesthesia, piglets underwent a surgical procedure for implanting catheters and probes.

The carotid artery was isolated by blunt dissection, and a catheter was inserted to allow arterial blood sampling. A jugular vein catheter was advanced to the superior vena cava to hydrate the pig throughout the procedure and to allow venous blood sampling. A laparotomy was then performed, the portal vein was isolated, and an ultrasonic perivascular blood flow probe (6 mm) (Transonic Systems Inc.) was secured around the portal vein to measure the blood flow. A catheter was also implanted in the portal vein for repeated blood sampling. A duodenal catheter was placed to infuse experimental diet treatments (described in experimental diets), and the exposed visceral organs were moistened with warmed saline and covered with wet gauze and plastic wrap during the surgery to prevent dehydration.

### Experimental diets

During the intraduodenal infusion, the following diet treatments were administered. Four treatments were as follows: *1)* GAA plus 20% methionine (20% Met); *2)* GAA plus 80% methionine (80% Met); *3)* GAA plus 140% methionine (140% Met); or *4)* GAA plus 200% methionine (200% Met). For these test infusions, methionine was added to provide different concentrations of methionine ranging from 2.08 to 20.77 mg·kg^−1^ BW·h^−1^; this represents 20%–200% of the methionine requirement with excess cysteine determined by Shoveller et al. [24]. GAA was added to the base dietary infusate to provide it at a rate of 3.75 mg·kg^−1^ BW·h^−1^. This amount of GAA, if completely converted to creatine, would fulfill the piglet's total creatine accretion rate at this age [13]. Methionine was delivered at a rate of 2.08, 8.30, 14.53, and 20.77 Met mg·kg^−1^ BW·h^−1^. [^3^H-methyl]-methionine (prime: 30 μCi·kg^−1^ BW; constant: 30 μCi·kg^−1^ BW·h^−1^) infusions (1 mL·kg^−1^ BW·h^−1^) were administered to trace the ^3^H-methyl incorporation into creatine.

All of the experimental diet treatments were made as previously described [23,25]. The test diets were completely elemental and were identical to each other except for methionine (test amino acid) and alanine (to balance nitrogen) (Supplemental Table 1). The complete diet, which was designed to supply all nutrients required by piglets, was delivered at 10 mL·kg^−1^ BW·h^−1^ to provide 13 g of amino acids·kg^−1^ BW·d^−1^ (Ajinomoto, Evonik Industries AG, Sigma Aldrich, or Bachem) and nonprotein energy of 0.55 MJ∙kg^-1^ BW·d^-1^ from dextrose (Sigma Aldrich). The solutions were sterile filtered via 0.22 μm filters (PALL Life Science) and stored in a cooler until used. All vitamins and minerals were supplied at >120% of the requirement for neonatal piglets [26]. Just before use, multivitamins, iron dextran (Bimeda-MTC Animal Health), and trace elements (Sigma Aldrich, Canada) were added to each diet, as previously described [27]. The experimental treatments were infused for 4 h. To achieve a postprandial state more quickly, a bolus priming dose of diet equal to the hourly rate was first infused, followed by continuous infusion at a rate of 11 mL·kg^−1^ BW·h^−1^via a syringe pump.

### Blood flow measurement and blood sampling

Blood samples from the portal vein and the carotid artery and portal blood flow rate measurements (mL·min^−1^) were obtained every 15 min for a total of 4 h. For the gut, blood flow from the portal vein was first measured 30 min after initiating the intraduodenal infusion. The blood flow was continuously recorded over a minimum of 2 min when the flow measurements' variability was <5% and those data were used to calculate the mean for that time point. During the treatment period (4 h), blood was sampled from the carotid artery (representing arterial metabolite concentrations available to the gut) and from the portal vein every 15 min. At the end of the experiment, tissues were removed (liver, kidneys, small intestine; section of mid jejunum, and biceps femoris muscle) and weighed (liver and kidneys). The piglets died by exsanguination after the removal of the organs, and tissue samples were immediately frozen in liquid nitrogen and stored at −80°C until analyzed.

### Metabolite analyses

#### Tissue and plasma GAA determination

Plasma and tissue GAA concentrations were measured by HPLC with ninhydrin derivatization and fluorescence detection [28]. The GAA concentration in plasma was measured using samples taken at 3 different time points: 3, 3.5, and 4 h. The mean value of these measurements was then utilized to determine the arteriovenous balance.

#### Tissue creatine concentration and specific radioactivity determination

Tissue creatine concentrations were measured by HPLC using a modified method by Lamarre et al. (2012) [29]. Tissue homogenates were deproteinized with trifluoroacetic acid after being homogenized in a 50 mmol·L^-1^ Tris buffer (pH 7.4) and left at room temperature for 20 min to allow for complete conversion of phosphocreatine to creatine. Creatine was separated with an isocratic mobile phase of 0.1% trifluoroacetic acid and 3% methanol. Then, creatine fractions were collected, and the radioactivity associated with these fractions was determined by liquid scintillation counting using Scintiverse (Fisher Chemical).

#### Tissue and plasma methionine determination

Plasma and tissue methionine concentrations were measured by HPLC following derivatization with phenylisothiocyanate with norleucine as the internal standard [30]. Plasma samples were first deproteinized with 0.5% trifluoroacetic acid in methanol. Tissue samples were homogenized in perchloric acid and the supernatant was used to determine free methionine concentration. Methionine fractions were collected during HPLC analysis and the radioactivity associated with these fractions was determined by liquid scintillation counting.

#### Arteriovenous balance

Net arteriovenous balance across the gut was determined by multiplying the concentration difference between venous and arterial blood (portal vein concentration minus arterial concentration) by the blood flow and correcting for body weight. The balance data were converted to percent change from the lowest methionine group (20% Met).

#### Calculations

The percent of dietary GAA that appeared in portal circulation was determined by dividing arteriovenous balance by dietary GAA infusion rate:

Portal GAA appearance (%) = arteriovenous GAA balance/GAA infusion rate ∗ 100

The fractional ^3^H-methyl incorporation (%) into creatine was determined using the following equation:

Fractional ^3^H-methyl incorporation (%) = (SRA product/SRA precursor) × 100,

where the specific radioactivity (SRA) product was measured as disintegrations per minute (DPM)·μmol^−1^for creatine and the SRA precursor was measured as DPM·μmol^−1^of tissue methionine.

### Statistical analyses

Data were analyzed using 1-way ANOVA followed by Duncan’s multiple range test to detect the differences between treatments. Polynomial contrasts were carried out to determine the linear and quadratic effects of increasing methionine concentrations (IBM SPSS Statistics; Version 27). Significant differences were recognized when the *P* value was <0.05. In addition, data were tested to verify whether they were significantly different from zero by 1-sample *t*-test (GraphPad Prism; Graph Pad Software Inc.).

## Results

### Jejunum GAA and creatine parameters

**FIGURE 1:**
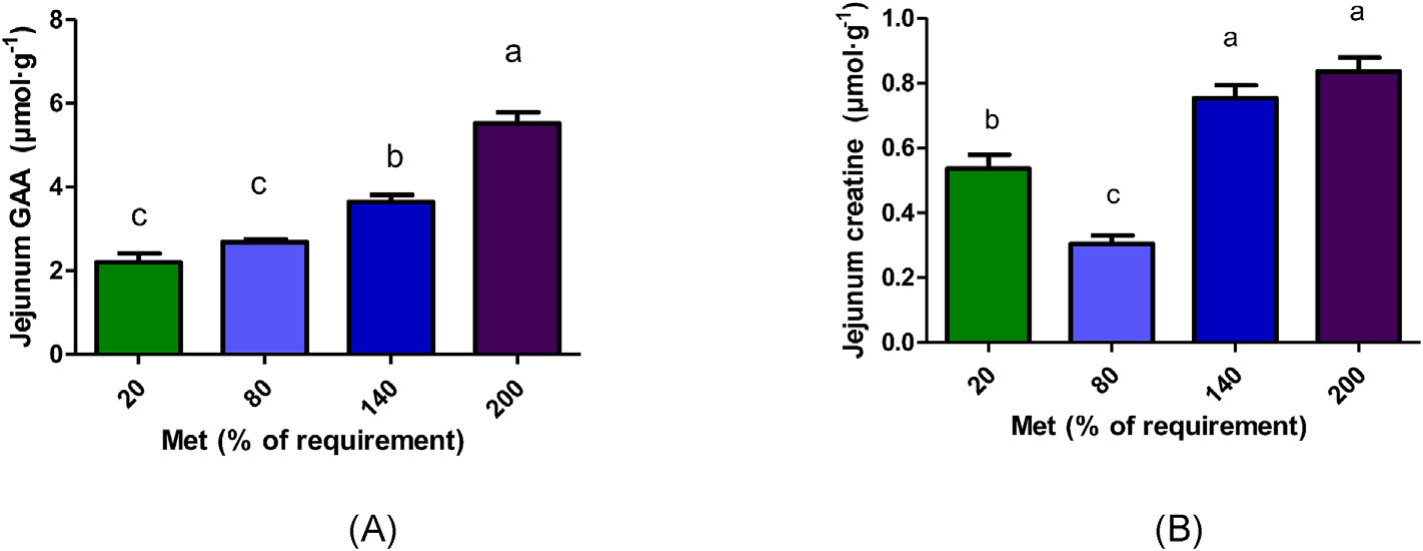
GAA (A) and creatine (B) concentrations in jejunum following 4 h duodenal infusion with diets containing 20% Met, 80% Met, 140% Met, or 200% Met in piglets. Values are means ± SEM; *P* values were calculated by ANOVA with polynomial contrasts for linear trend. Bars with different letters are significantly different (*P* < 0.05). (A) *P* value for Trt < 0.001, *P* value for L < 0.001; (B) *P* value for Trt < 0.001, *P* value for L < 0.001. GAA, guanidinoacetic acid; L, linear effect; Met, methionine; Trt, treatment effect.

The ANOVA showed that dietary methionine had a significant effect on GAA and creatine concentrations and the fractional ^3^H-methyl incorporation into creatine in jejunum. Moreover, polynomial contrasts showed, with increasing methionine concentrations, jejunum GAA and creatine concentrations, and ^3^H-methyl incorporation into creatine increased linearly (*P* < 0.001; Table 1, Supplemental Tables 2 and 3). GAA concentration in jejunal mucosa was significantly higher in piglets infused with 200% Met treatment (*P* < 0.001; Figure 1A). The jejunum creatine concentration (*P* < 0.001; Figure 1B) and the fractional ^3^H-methyl incorporation into creatine (*P* < 0.001; Table 1) were also significantly higher in the 200% and 140% Met groups.

**TABLE 1:** Fractional ^3^H methyl incorporation (%) into creatine in piglets given 4 h duodenal infusion with diets containing 20% Met, 80% Met 140% Met, or 200% Met.

Fractional ^3^H methyl incorporation (%) into creatine	Treatments					*P* value		
	20% Met	80% Met	140% Met	200% Met	SEM	Trt	L	Q
Jejunum (%)	14.94^1^	22.04^1^	90.00^2^	114.14^2^	7.08	<0.001	<0.001	0.556
Liver (%)	17.22^1^	31.70^1^	81.76^2^	41.05^1^	5.00	0.002	0.018	0.015
Kidney (%)	0.90	0.81	1.39	1.36	0.15	0.386	0.167	0.294
Muscle (%)	1.95^2^	0.52^1^	0.37^1^	0.69^1^	0.08	<0.001	<0.001	<0.001

BW, body weight; L, linear effect; Q, quadratic effect; Trt, treatment effect. Met, methionine; 20% Met = 2.08 mg·kg^−1^ BW·h^−1^, 80% Met = 8.3 mg·kg^−1^ BW·h^−1^, 120% Met = 14.53 mg·kg^−1^ BW·h^−1^, 200% Met = 20.77 mg·kg^−1^ BW·h^−1^.

^1–^^2^Differences between treatments (*P* < 0.05).

### Plasma GAA and creatine concentrations

**FIGURE 2:**
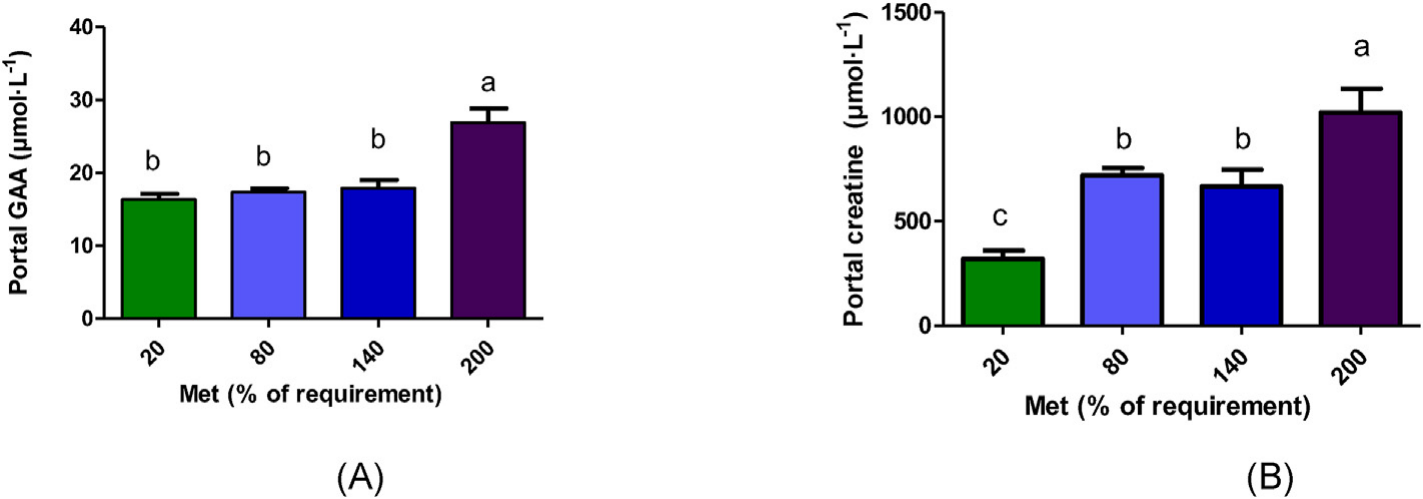
GAA (A) and creatine (B) concentrations in portal vein following 4 h duodenal infusion with diets containing 20% Met, 80% Met, 140% Met, or 200% Met in piglets. Values are means ± SEM; *P* values were calculated by ANOVA with polynomial contrasts for linear trend. Bars with different letters are significantly different (*P* < 0.05). (A) *P* value for Trt < 0.001, *P* value for L < 0.001; (B) *P* value for Trt < 0.001, *P* value for L < 0.001. GAA, guanidinoacetic acid; L, linear effect; Met, methionine; Trt, treatment effect.

**FIGURE 3:**
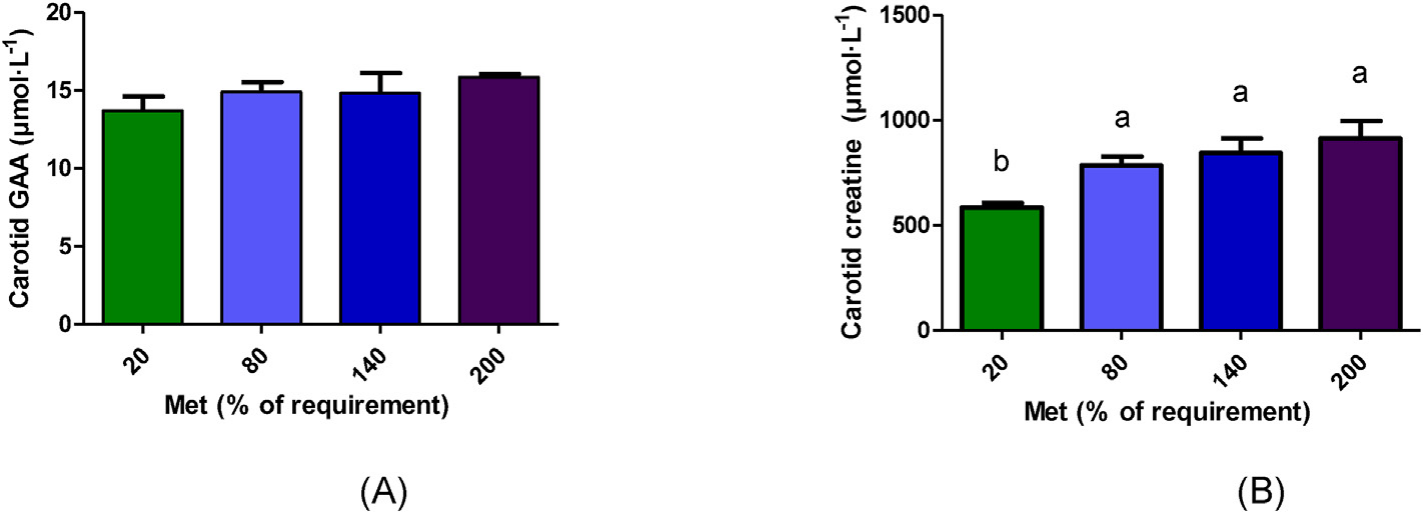
Guanidinoacetic acid (A) and creatine (B) concentrations in carotid artery following 4 h duodenal infusion with diets containing 20% Met, 80% Met 140% Met, or 200% Met in piglets. Values are means ± SEM; *P* values were calculated by ANOVA with polynomial contrasts for linear trend. ANOVA. Bars with different letters are significantly different (*P* < 0.05). (A) *P* value for Trt = 0.398; *P* value for L = 0.116 and (B) *P* value for Trt = 0.017, *P* value for L = 0.002. L, linear effect; Met, methionine; Trt, treatment effect.

Plasma (portal and carotid) creatine and portal GAA concentrations were significantly different across various methionine concentrations (FIGURE 2, FIGURE 3B). However, carotid GAA concentration was not significantly different among the groups (*P* > 0.05; Figure 3A). According to polynomial contrasts, portal GAA and creatine concentrations (*P* < 0.001) and carotid creatine concentration (*P* = 0.002) increased linearly with increasing methionine concentrations (Supplemental Tables 2 and 3). GAA and creatine concentrations in the portal vein samples were significantly higher in the 200% Met group (*P* < 0.001; Figure 2). Carotid creatine concentration was significantly higher in the 80%–200% Met groups compared with the 20% Met group (*P* = 0.017; Figure 3B).

### Gut GAA and creatine balance

**FIGURE 4:**
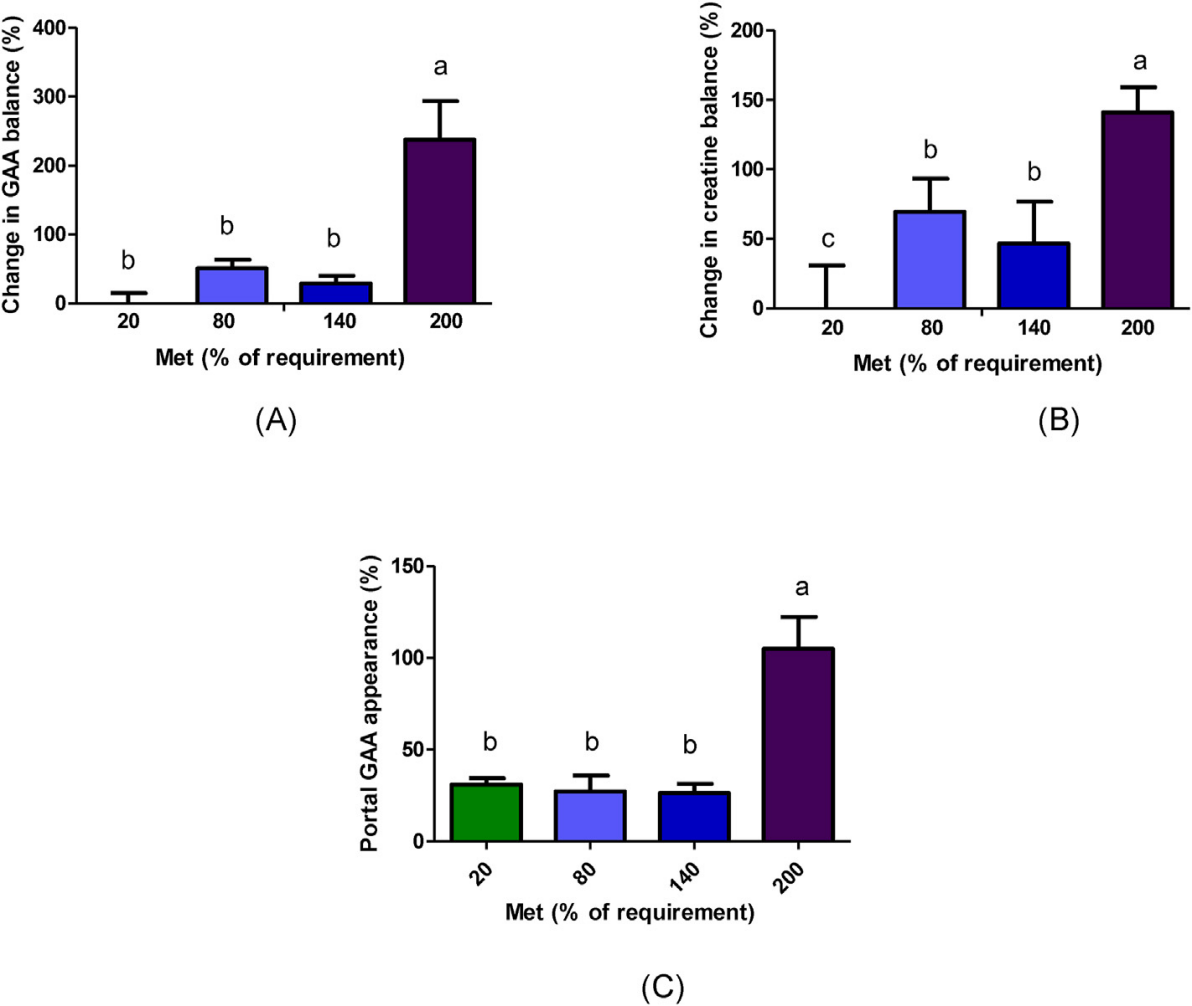
Change in portal GAA balance percentage (A), change in creatine balance percentage (B), and portal GAA appearance (C) during 4 h duodenal infusion with diets containing 20% Met, 80% Met 140%, Met, or 200% Met in piglets. Values are means ± SEM; *P* values were calculated by ANOVA with polynomial contrasts for linear trend. Bars with different letters are significantly different (*P* < 0.05). (A) *P* value for Trt < 0.001, *P* value for L < 0.001; (B) *P* value for Trt < 0.001, *P* value for L < 0.001; (C) *P* value for Trt < 0.001, *P* value for L < 0.001. GAA, guanidinoacetic acid; L, linear effect; Met, methionine; Trt, treatment effect.

The ANOVA results revealed significant differences in change in GAA portal balance and GAA appearance with increasing methionine concentrations. Furthermore, there was a clear linear increase in GAA appearance as methionine concentrations increased (*P* < 0.001; Supplemental Table 2). The 200% Met group had a significantly greater change in GAA balance compared with 20%–140% Met groups (*P* < 0.001; Figure 4A). GAA appearance in the portal vein was significantly higher in the 200% Met group (104.9%) compared with 20%, 80%, and 140% Met groups (31.1%, 27.4%, 26.3%, respectively) (*P* < 0.001; Figure 4C, Supplemental Table 2).

The change in creatine portal balance also exhibited significant differences among the various groups with different concentrations of methionine. The 200% Met group demonstrated a significantly greater change in creatine balance across the gut, compared with the 20% Met group (*P* < 0.001; Figure 4B)

### Liver GAA and creatine parameters

**FIGURE 5:**
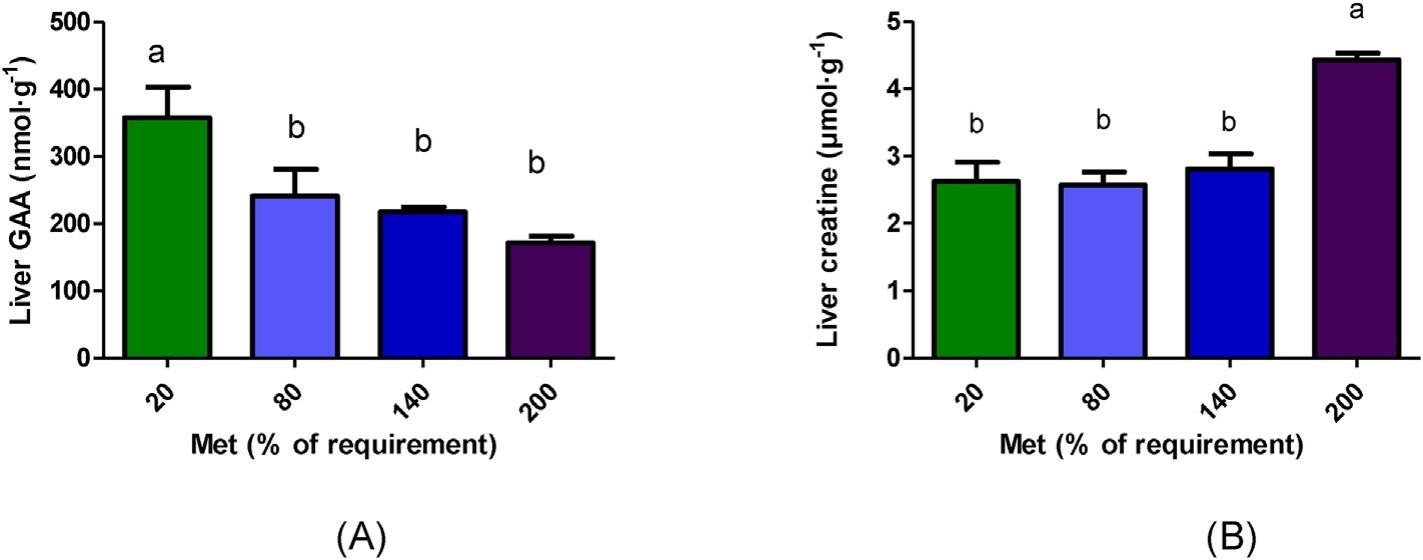
GAA (A) and creatine (B) concentrations in the liver following 4 h duodenal infusion with diets containing 20% Met, 80% Met 140% Met, or 200% Met in piglets. Values are means ± SEM; *P* values were calculated by ANOVA with polynomial contrasts for linear trend. Bars with different letters are significantly different (*P* < 0.05). (A) *P* value for Trt = 0.004, *P* value for L < 0.001; (B) *P* value for Trt < 0.001, *P* value for L < 0.001. GAA, guanidinoacetic acid; Met, methionine; L, linear effect; Trt, treatment effect.

According to the ANOVA analysis, there were significant changes in liver GAA and creatine concentrations in response to varying concentrations of methionine. Liver GAA concentration decreased linearly with increasing methionine concentrations (*P* < 0.001; Supplemental Table 2). Creatine concentration (*P* < 0.001; Supplemental Table 2) and fractional ^3^H-methyl incorporation into creatine (*P* = 0.018; Table 1) increased with the methionine concentration. Hepatic GAA concentration was significantly higher after the 20% Met treatment; but it was not significantly different among the 80%–200% Met groups (*P* = 0.004; Figure 5A). Hepatic creatine concentration (*P* < 0.001; Figure 5B) and fractional ^3^H-methyl incorporation into creatine (*P* = 0.002; Table 1) were significantly greater in 200% and 140% Met groups, respectively, compared with other treatment groups.

### Kidney GAA and creatine parameters

**FIGURE 6:**
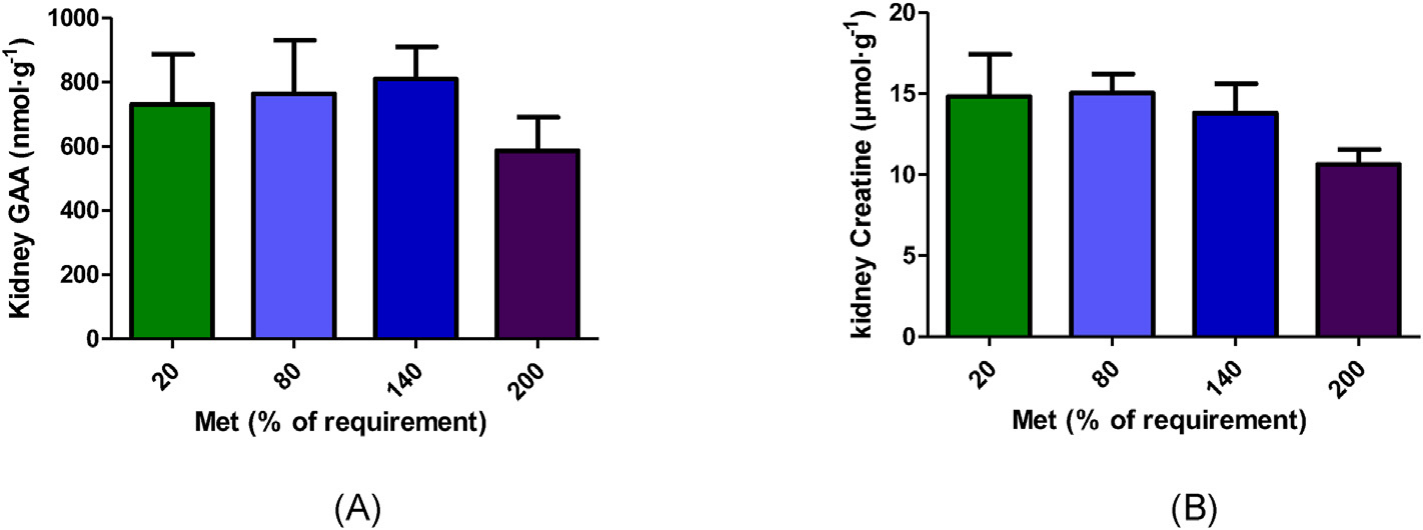
Guanidinoacetic acid (A) and creatine (B) concentration in the kidney following 4 h duodenal infusion with diets containing 20% Met, 80% Met 140% Met, or 200% Met in piglets. Values are means ± SEM; *P* values were calculated by ANOVA with polynomial contrasts for linear trend. (A) *P* value for Trt = 0.824, *P* value for L = 0.515; (B) *P* value for Trt = 0.069, *P* value for L = 0.032. L, linear effect; Met, methionine; Trt, treatment effect.

Dietary methionine concentrations did not impact the GAA and creatine concentrations in the kidney (*P* > 0.05; Figure 6). Creatine SRA was significantly higher in the 200% Met group than in other groups (*P* < 0.001; Table 2); however, the fractional ^3^H-methyl incorporation into creatine was not significantly different among the groups (*P* > 0.05; Table 1).

**TABLE 2:** Creatine SRA in tissues after 4 h duodenal infusion with diets containing 20% Met, 80% Met, 140% Met, or 200% Met in piglets.

Creatine SRA	Treatments					*P* value		
	20% Met	80% Met	140% Met	200% Met	SEM	Trt	L	Q
Jejunum creatine SRA (DPM·μmol^−1^)	11,416^1^	23,307^2^	35,978^3^	32,078^3^	1774	<0.001	<0.001	0.041
Liver creatine SRA (DPM·μmol^−1^)	7201^1^	5610^1^	25,394^3^	15,348^2^	1034	<0.001	<0.001	0.058
Kidney creatine SRA (DPM·μmol^−1^)	559^2^	514^2^	555^2^	866^3^	19	<0.001	<0.001	<0.001
Muscle creatine SRA (DPM·μmol^−1^)	271	249	231	203	25	0.795	0.328	0.945
Carotid creatine SRA (DPM·μmol^−1^)	12,803	12,613	10,378	11,756	886	0.763	0.507	0.664

BW, body weight; DPM, disintegrations per minute; L, linear effect; Q, quadratic effect; Trt, treatment effect; SRA, specific radioactivity. Met, methionine; 20% Met = 2.08 mg·kg^−1^ BW·h^−1^, 80% Met = 8.3 mg·kg^−1^ BW·h^−1^, 120% Met = 14.53 mg·kg^−1^ BW·h^−1^, 200% Met = 20.77 mg·kg^−1^ BW·h^−1^.

^1–3^ Differences between treatments (*P* < 0.05).

### Muscle creatine parameters

**FIGURE 7:**
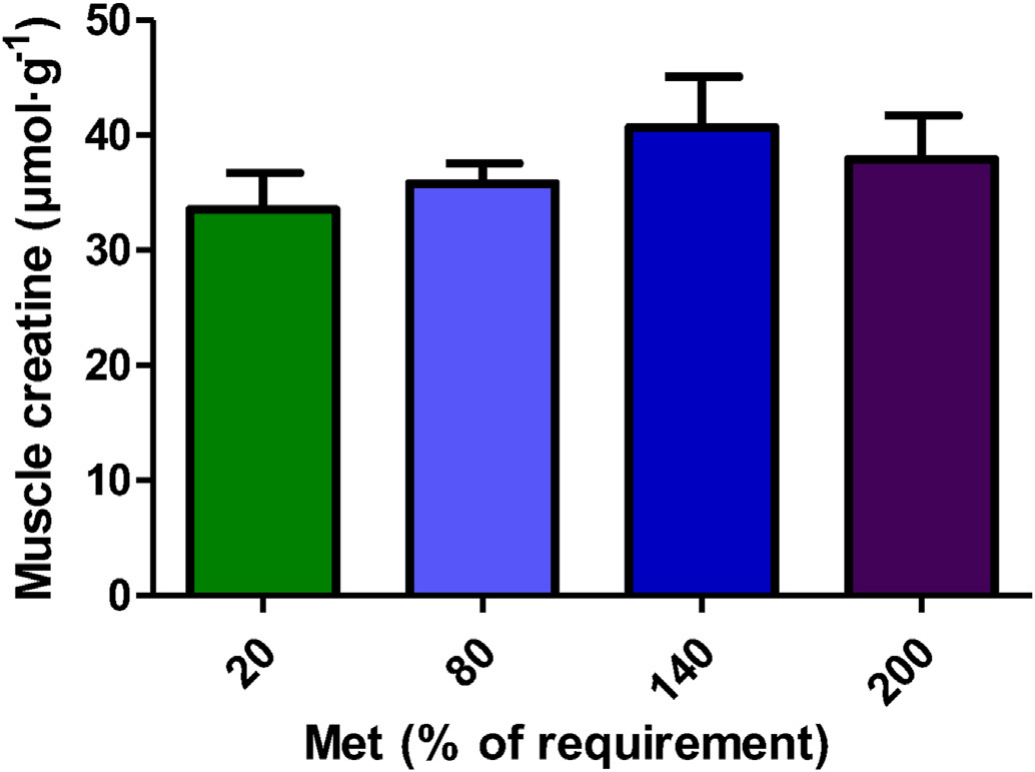
Creatine concentration in muscle following 4 h duodenal infusion with diets containing 20% Met, 80% Met 140% Met, or 200% Met in piglets. Values are means ± SEM; *P* values were calculated by ANOVA with polynomial contrasts for linear trend. *P* value for Trt = 0.522; *P* value for L = 0.258. L, linear effect; Met, methionine; Trt, treatment effect.

We were unable to observe any increase in muscle creatine during the 4 h infusion; as shown in Figure 7, muscle creatine concentration was not significantly different among the groups (*P* > 0.05). However, with increasing methionine concentrations, fractional ^3^H-methyl incorporation into creatine increased linearly (*P* < 0.001; Table 1). The fractional ^3^H-methyl incorporation into creatine in muscle was significantly higher in the 20% Met group (*P* < 0.001; Table 1) compared with the other groups.

### Carotid creatine SRA

Carotid creatine SRA was not significantly different among the Met groups (*P* > 0.05; Table 2).

### Methionine concentrations in plasma and tissues

The concentrations of methionine in both plasma and tissues displayed significant changes in response to varying concentrations of methionine intake. Polynomial contrasts showed, with increasing methionine concentration, tissue and plasma methionine concentrations increased linearly (*P* < 0.05; Table 3). At the end of the 4 h infusion, methionine concentrations in the portal vein, carotid artery, kidney, liver, jejunum, and muscle were significantly higher in the 200% Met group; for jejunum, kidney, liver, and portal vein, 140% Met group also had significantly higher methionine concentrations than the lower Met groups (*P* < 0.05; Table 3).

**TABLE 3:** Plasma and tissue methionine concentrations in piglets given 4 h duodenal infusion with diets containing 20% Met, 80% Met, 140% Met, or 200% Met.

Methionine concentration	Treatments					*P* value		
	20% Met	80% Met	140% Met	200% Met	SEM	Trt	L	Q
Jejunum methionine (nmol·g^−1^)	580^1^	669^1^	1115^2^	1434^2^	54	<0.001	<0.001	0.302
Kidney methionine (nmol·g^−1^)	144^1^	179 ^1^	275^2^	274^2^	11	<0.001	<0.001	0.417
Liver methionine (nmol·g^−1^)	88^1^	192^2^	233^2^	182^2^	12	<0.001	0.011	0.007
Muscle methionine (nmol·g^−1^)	258^1^	209^1^	285^1^	615^2^	33	0.002	0.001	0.011
Portal methionine (μmol·L^−1^)	151^1^	313^1^	447^2^	520^2^	31	0.003	<0.001	0.483
Carotid methionine (μmol·L^−1^)	122^1^	153^1^	221^1^	326^2^	17	0.004	<0.001	0.3

BW, body weight; L, linear effect; Q, quadratic effect; Trt, treatment effect. Met, methionine; 20% Met = 2.08 mg·kg^−1^ BW·h^−1^, 80% Met = 8.3 mg·kg^−1^ BW·h^−1^, 120% Met = 14.53 mg·kg^−1^ BW·h^−1^, 200% Met = 20.77 mg·kg^−1^ BW·h^−1^

^1–^^2^ Differences between treatments (*P* < 0.05).

## Discussion

GAA is considered a novel supplement to restore creatine and enhance growth performance in the commercial animal industry [31,32]. The supplement is also being investigated in humans, and studies have evaluated the effects of dietary GAA as an alternative to creatine supplements [1]. To achieve the optimum efficacy of exogenous GAA, it must be absorbed by the small intestine and converted into creatine in various target tissues, using methionine as a methyl donor. In this study in neonatal piglets, we demonstrated that the efficacy of GAA supplements is dependent on the amount of dietary methionine present with GAA.

This research question arose from a previous study in our laboratory in which piglets fed a diet supplemented with GAA, but with limited dietary methionine (80% of requirement), had limited creatine synthesis, compared with when sufficient methionine was fed [18]. Yet, despite this limited GAA conversion to creatine, GAA did not appear to accumulate in plasma, liver, muscle, kidney, jejunum, or brain [18]. If dietary GAA was not utilized for creatine synthesis and did not sequester in key organs, then presumably it must have been either rapidly excreted via the kidney, reversibly metabolized to arginine by AGAT, or was not absorbed when methionine was deficient. In those previous studies, we did not observe higher urinary GAA concentrations, nor did we observe an increase in arginine concentrations; so, we hypothesized that GAA absorption was dependent on dietary methionine availability. Therefore, this study aimed to describe the fate of supplemental GAA in neonatal piglets over a range of dietary methionine intakes. In the current study, we demonstrated that insufficient dietary methionine limits dietary GAA absorption, lowers creatine synthesis in the liver, kidney, and jejunal mucosa, and leads to the accumulation of GAA in the liver.

According to the current study, dietary methionine significantly impacted GAA absorption in piglets. However, the mechanistic relationship between dietary methionine and GAA absorption is unknown. The present study showed that 140% Met and 200% Met groups resulted in the highest jejunum concentration of GAA. These data suggest that GAA absorption was enhanced by dietary methionine. Despite this indirect evidence, there is not much known regarding GAA absorption across the gut in animals and humans. GAA supplements in chickens have 98%–99% fecal digestibility, which is not affected by the concentration of supplemental GAA [33]. However, the role of methionine in this digestibility assessment was not addressed. The current findings in piglets underscore the significance of having a sufficient supply of methionine to enhance complete GAA absorption across the gut. Notably, the group with 200% methionine exhibited a 105% portal GAA appearance, indicating the crucial role of an adequate methionine supply in promoting optimal GAA absorption; these data likely represent GAA absorption, because negligible amounts of GAA are synthesized de novo and released by the gut to the circulation [14]. Surprisingly, the pigs administered 140% methionine, which is still above the estimated methionine requirement, had only ∼26% portal GAA appearance, similar to that in pigs fed deficient concentrations of methionine. We speculate that the remaining ∼74% might either not be completely absorbed by the gut, utilized within the gut for creatine synthesis, or metabolized by gut microbes. Furthermore, the current study provides some of the first evidence on GAA absorption and its utilization toward its primary product, creatine, and how dietary methionine can alter these processes in piglets.

GAA transport is thought to occur through the creatine transporter (CRT/Solute carrier SLC6A8), taurine transporters (TauT/SLC6A6), γ-aminobutyric acid transporter (SLC6 A13), and passive diffusion through plasmalemma [34]. Supplementing GAA with creatine may lead to saturation of creatine transporters, increasing GAA transport via other transporters, resulting in greater total GAA and creatine concentrations in cells [35]. Intestinal transport of methionine mainly occurs through Na^+^-independent or -dependent transporters. The apical transport systems are Na^+^-dependent, and on the basolateral side, methionine efflux is facilitated by the Na^+^-independent system [36]. Because methionine and GAA use different transporters, there is no obvious competition or synergic mechanism at the transporter site. A more likely explanation is that when dietary methionine is abundant, GAA is transported and rapidly converted to creatine. This synthesis of creatine lowers GAA concentrations, maintaining a concentration gradient for GAA transport. Indeed, creatine concentrations in most tissues and blood increased in parallel with methionine concentrations. Therefore, when GAA is supplemented with excess methionine, it may increase GAA absorption and subsequently promote its conversion into creatine.

Although GAA transport from the lumen into the jejunum was higher in both 140% Met and 200% Met groups, GAA release to the portal vein was significantly higher only in the 200% Met group. Indeed, although the net absorption of GAA was over 100% in the 200% Met group, only 26%–31% of dietary GAA appeared in the portal vein for the 3 lower Met groups. This finding aligns with the study by Dinesh et al. (2020) [14], which suggested that GAA is released from the gut only when there is a sufficient amount of circulating creatine in the body. In the current study, it is also notable that the portal vein creatine concentrations were lower than the carotid artery concentrations for all groups except 200% Met. These data suggest that creatine is extracted from the circulation by the gut in the 20%, 80%, and 140% Met groups; but in the 200% Met group, creatine and GAA are released by the gut to the circulation. In other words, when dietary methionine is low, the gut retains most dietary GAA, but also extracts creatine from the circulation, presumably because methionine is limiting GAA conversion to creatine in the gut. With excess dietary methionine (200% Met group), the gut appears to transport dietary GAA to the portal circulation. Overall, excess dietary methionine was needed for GAA absorption from the lumen to the portal vein, which has the additional effect of increasing creatine release to the circulation.

Alternatively, there may be another reason behind the finding of the high amount of GAA in the jejunum with the presence of 200% methionine. The gut releases a significant amount of endogenously synthesized GAA, when blood creatine is abundant [14]. This appears contradictory to our previous finding of undetectable concentrations (<10 nmol·min^−1^·g^−1^) of intestinal AGAT; however, the total small intestine (∼120 g) may still have AGAT capacity to produce ≤1200 nmol/min of GAA, which can be significant to whole-body creatine homeostasis. This capacity is ∼32% of kidney AGAT capacity (211 nmol·min^−1^·g^−1^ × 18 g = ∼3800 nmol/min) [13], so more work needs to be done on small intestinal creatine metabolism. We previously found that there is a net release of GAA from the gut, but only when there is excess circulating creatine in the body [14].

As methionine is increased in the diet, creatine balance across the gut increases, such that in the highest Met group, the increase reached ∼140%. This underscores how elevated methionine concentrations promote the conversion of GAA to creatine, resulting in an increased creatine portal balance in piglets. Likewise, there was a noteworthy increase in the percentage change of GAA portal balance in the 200% Met group. This highlights the influence of methionine on GAA portal balance, although the underlying mechanism remains incompletely understood.

We hypothesized that when circulating creatine is low, the gut synthesizes creatine from GAA to be used locally; but when creatine is readily available, GAA synthesized by the gut is released to the circulation for creatine biosynthesis elsewhere. In the current study, when excess dietary methionine was available, creatine biosynthesis was induced and abundant, which increased circulating creatine concentrations. Consistent with our previous study, any unused GAA accumulated in the gut and was released into the circulation. In contrast, when circulating creatine is limited, as in the lower methionine groups, GAA synthesized by the gut was used locally for creatine biosynthesis.

Consistent with this hypothesis, higher dietary methionine with supplemental GAA increased jejunum creatine synthesis, supporting our previous data suggesting the gut is an important site of creatine synthesis, but only with sufficient dietary methionine [14]. This is further supported by our finding of intermediate concentrations of GAMT activity in the piglet jejunum [13]. Consistent with this, fractional ^3^H-methyl incorporation into creatine in jejunum was highest in high methionine groups emphasizing that a sufficient amount of methionine is required to synthesize creatine and improve creatine pools in the body. The fate of this locally synthesized creatine in the jejunum is unknown, as it can be used by the gut or transported to other tissues. However, the creatine balance data suggest that there is a net release of creatine from the gut only when methionine is in excess.

Creatine synthesis in the liver was also enhanced by the higher concentrations of methionine, underlining that the transmethylation of GAA to creatine was active in the liver and dependent on adequate concentrations of dietary methionine. The current study results show higher creatine concentration in the 140% Met group compared with the 200% Met group. Within the 200% Met group, an increased synthesis of creatine occurs due to the surplus methionine, leading to an abundance of creatine available for liver uptake and utilization. However, this surplus of available creatine might also slow down further creatine production (via renal AGAT) and/or facilitate greater transport to other tissues compared with the 140% group. Consequently, the liver may end up retaining a lower amount of creatine when methionine is in excess (200% Met group). The portal vein transports the absorbed GAA to the liver, where hepatic GAMT activity is highest [13], and converts GAA to creatine using methyl groups from methionine. Therefore, the higher creatine concentration and fractional ^3^H-methyl incorporation into creatine in 200% and 140% Met groups demonstrate that more dietary methionine facilitates GAA conversion to creatine in the liver. Interestingly, this effect was stimulated acutely by luminal methionine, suggesting intrahepatic sources of methionine are not sufficient to maintain this conversion to creatine. Consequently, hepatic GAA concentrations decreased with increasing dietary methionine concentrations, as GAA is increasingly consumed for creatine synthesis.

In contrast, creatine synthesis was lowest in the 20% Met group, suggesting methyl groups were limiting for the transmethylation of GAA to creatine. Therefore, GAA accumulated in the liver of 20% Met piglets, as it was not used for creatine synthesis. Of the tissues measured, the most profound difference in GAA accumulation was observed in the liver, reflecting its central role in whole-body creatine synthesis. We have previously determined that there is no measurable AGAT activity in the piglet liver [13]. This means that the accumulated GAA in the liver originated from either the absorbed supplemental GAA or endogenous GAA transported via circulation. In either case, the accumulation of unused GAA in some tissues could be dangerous [37]. However, so far, no studies have reported an incidence of neurotoxicity after dietary GAA supplementation in humans or pigs.

High-dietary methionine also enhanced renal creatine synthesis with GAA supplementation. Although GAA and creatine concentrations in the kidney were not significantly different among the groups, creatine SRA was highest in the 200% Met group. These data suggest that newly synthesized creatine was transported to the kidney or was produced by the kidney as more methyl groups are available in the highest methionine group. Renal GAMT activity is significant in the piglet so the kidney is capable of complete biosynthesis of GAA and creatine [13]. The rapid conversion of GAA to creatine in the kidney is supported by the fact that GAA did not accumulate in the kidney. Alternatively, newly synthesized GAA was rapidly transported to the plasma for other tissues to utilize for extrarenal creatine synthesis. Moreover, arterial (carotid) creatine SRA was not significantly different among groups, emphasizing that the differences in tissue fractional ^3^H methyl incorporation were more likely due to the new creatine synthesis in individual tissues.

Our short infusion time was not sufficient to detect any changes in muscle creatine concentrations. Because muscle has no detectable AGAT activity and very low GAMT activity, newly synthesized creatine may have been transported from the liver. Although we expected a higher concentration of creatine in the 200% Met group, our short experimental period likely did not allow us to detect changes in this very large pool of creatine [12]. In a long-term feeding study in ducks, supplemental GAA with methionine enhanced muscle creatine loading capacity [6]. So, it is possible that with a longer infusion time, muscle creatine concentrations could be increased.

In summary, the results from this study demonstrated that when piglets were supplemented with GAA and excess methionine, enhanced creatine synthesis in various tissues occurred. When dietary methionine was low, supplemented GAA was not absorbed, and what was absorbed accumulated in the liver. Although the gut is known to synthesize creatine, the fate and role of this creatine are not well investigated. Some studies have shown the beneficial effects of creatine in the gut. For example, a few human case reports have demonstrated that dietary creatine attenuates inflammatory bowel disease by maintaining gut barrier function [38]. The current study indicates that GAA with sufficient methionine enhances creatine pools in the jejunum, which may be a beneficial alternative to creatine supplements in maintaining gut functions. Besides its role in the gut, it has been demonstrated that GAA is a beneficial and safe dietary supplement in humans and animals. Among its many demonstrated benefits, GAA, likely via conversion to creatine, promotes growth, physical performance, reproductive parameters, and meat quality in commercial animals [10,11].

In conclusion, the findings from this study demonstrated that in neonatal piglets, excess methionine is required to achieve 100% GAA absorption. However, the results of this study cannot define the methionine requirement for GAA absorption across the gut. Nevertheless, creatine stores were superior in piglets that received methionine at approximately twice the estimated whole-body methionine requirement. However, caution is warranted before recommending doubling of methionine requirements, given its potential role in increasing homocysteine concentrations, a well-known cardiovascular disease risk factor. In conclusion, it is clear that when using GAA supplements, sufficient dietary methionine should also be administered to enhance complete GAA absorption and creatine synthesis in neonatal piglets.

### Author contributions

The authors’ responsibilities were as follows – MA, CD, JB, RB: designed research; MA, CD: conducted research; MA: analyzed samples and performed statistical analyses; MA, RB: wrote the manuscript and had primary responsibility for the final content; and all authors: were responsible for designing the study, contributed to the manuscript, and read and approved the final manuscript.

### Funding

This research was funded by the Natural Sciences and Engineering Research Council of Canada.

### Conflict of interest

The authors report no conflicts of interest.
