# Supplementary material for: Dietary Methionine Enhances Portal Appearance of Guanidinoacetate and Synthesis of Creatine in Yucatan Miniature Piglets
Source: J Nutr. 2024 Mar 26;154(5):1571–81. doi: 10.1016/j.tjnut.2024.03.017 (PMC11130667; doi:10.1016/j.tjnut.2024.03.017)
Supplement: Multimedia component 1 [file mmc1.docx]

**Supplemental Table 1**. Amino acid and GAA concentration in the dietary infusates.

|  | **Dietary Infusate** | | | |
| --- | --- | --- | --- | --- |
| **Amino Acids** | **20% Met** | **80% Met** | **140% Met** | **200% Met** |
|  | **g·L^-1^** | | | |
| Alanine | 6.39 | 6.02 | 5.64 | 5.27 |
| Arginine | 3.65 | 3.65 | 3.65 | 3.65 |
| Aspartic Acid | 3.32 | 3.32 | 3.32 | 3.32 |
| Cysteine | 0.76 | 0.76 | 0.76 | 0.76 |
| Glutamic Acid | 5.72 | 5.72 | 5.72 | 5.72 |
| Glycine | 1.47 | 1.47 | 1.47 | 1.47 |
| Histidine | 1.69 | 1.69 | 1.69 | 1.69 |
| Isoleucine | 2.51 | 2.51 | 2.51 | 2.51 |
| Leucine | 5.67 | 5.67 | 5.67 | 5.67 |
| Lysine-HCl | 5.58 | 5.58 | 5.58 | 5.58 |
| Methionine^1^ | 0.21 | 0.83 | 1.45 | 2.08 |
| Phenylalanine | 3.00 | 3.00 | 3.00 | 3.00 |
| Proline | 4.52 | 4.52 | 4.52 | 4.52 |
| Serine | 3.11 | 3.11 | 3.11 | 3.11 |
| Taurine | 0.27 | 0.27 | 0.27 | 0.27 |
| Tryptophan | 1.14 | 1.14 | 1.14 | 1.14 |
| Tyrosine | 0.44 | 0.44 | 0.44 | 0.44 |
| Valine | 2.89 | 2.89 | 2.89 | 2.89 |
| Threonine | 2.23 | 2.23 | 2.23 | 2.23 |
| GAA^2^ | 0.375 | 0.375 | 0.375 | 0.375 |

^1^Methionine in the dietary infusate was provided at 20%. 80%, 140% or 200% of the requirement (2.08-20.77 mg·kg^−1^ BW·h^−1^) as determined by Shoveller et al., 2003 (24) for young pigs. Alanine concentration was manipulated to provide isonitrogenicity, with respect to amino acids. GAA, guanidinoacetic acid.

^2^GAA was provided at a rate of 3.75 mg·kg^−1^ BW·h^−1^: this amount of GAA, if completely converted to creatine, would fulfill the piglet's total creatine accretion rate at this age (13).

**Supplemental Table 2**- GAA concentration in tissues after 4 h duodenal infusion with diets containing 20% Met, 80% Met, 140% Met, or 200% Met in piglets.

|  | **Treatments** | | | | | **P-value^1^** | | |
| --- | --- | --- | --- | --- | --- | --- | --- | --- |
| **GAA** | **20% Met** | **80% Met** | **140% Met** | **200% Met** | **SEM** | **Trt** | **L** | **Q** |
| Jejunum GAA (µmol·g^-1^) | 2.20^c^ | 2.68^c^ | 3.64^b^ | 5.52^a^ | 0.10 | <0.001 | <0.001 | 0.002 |
| Liver GAA (nmol·g^-1^) | 357.75^a^ | 241.01^b^ | 217.42^b^ | 171.62^b^ | 14.49 | 0.004 | <0.001 | 0.240 |
| Kidney GAA  (nmol·g^-1^) | 792.92 | 804.86 | 815.78 | 634.14 | 78.09 | 0.824 | 0.515 | 0.544 |
| Carotid GAA (µmol·L^-1^) | 13.71 | 14.90 | 14.82 | 15.83 | 0.42 | 0.398 | 0.116 | 0.919 |
| Portal GAA (µmol·L^-1^) | 16.39^b^ | 17.35^b^ | 17.93^b^ | 26.92^a^ | 0.61 | <0.001 | <0.001 | 0.005 |
| Change in GAA portal balance (%) | 0^b^ | 50.91^b^ | 29.03^b^ | 237.6^a^ | 30.05 | <0.001 | <0.001 | 0.022 |
| Portal GAA appearance (%) | 31.08^b^ | 27.37^b^ | 26.31^b^ | 104.93^a^ | 5.07 | <0.001 | <0.001 | <0.001 |

^1^ L, linear effect; Q, quadratic effect; Trt, treatment effect.

^a–c^ Differences between treatments (P < 0.05).

SEM, standard error of the mean

Met, methionine; 20% Met = 2.08 mg·kg^−1^ BW·h^−1^, 80% Met= 8.3 mg·kg^−1^ BW·h^−1^, 120% Met=14.53 mg·kg^−1^ BW·h^−1^, 200% Met=20.77 mg·kg^−1^ BW·h^−1^

**Supplemental Table 3**- creatine concentration in tissues after 4 h duodenal infusion with diets containing 20% Met, 80% Met, 140% Met, or 200% Met in piglets.

|  | **Treatments** | | | |  | **P-value^1^** | | |
| --- | --- | --- | --- | --- | --- | --- | --- | --- |
| **Creatine** | **20% Met** | **80% Met** | **140% Met** | **200% Met** | **SEM** | **Trt** | **L** | **Q** |
| Jejunum creatine (µmol·g^-1^) | 0.53^b^ | 0.30^c^ | 0.76^a^ | 0.84^a^ | 0.04 | <0.001 | <0.001 | 0.001 |
| Liver creatine (µmol·g^-1^) | 2.62^b^ | 2.57^b^ | 2.81^b^ | 4.43^a^ | 0.21 | <0.001 | <0.001 | 0.001 |
| Kidney creatine  (µmol·g^-1^) | 14.80 | 15.03 | 13.78 | 10.63 | 1.76 | 0.069 | 0.032 | 0.107 |
| Muscle creatine (µmol·g^-1^) | 33.55 | 35.79 | 40.68 | 37.99 | 3.44 | 0.522 | 0.258 | 0.48 |
| Carotid creatine (µmol·L^-1^) | 586.01^b^ | 784.79^a^ | 843.32^a^ | 913.03^a^ | 31.10 | 0.017 | 0.002 | 0.316 |
| Portal creatine (µmol·L^-1^) | 318.85^c^ | 719.56^b^ | 665.65^b^ | 1020.14^a^ | 41.56 | <0.001 | <0.001 | 0.785 |
| Change in creatine portal balance (%) | 0^c^ | 69.47^b^ | 46.61^b^ | 140.90^a^ | 4.74 | <0.001 | <0.001 | 0.209 |

^1^ L, linear effect; Q, quadratic effect; Trt, treatment effect.

^a–c^ Differences between treatments (P < 0.05).

SEM, standard error of the mean

Met, methionine; 20% Met = 2.08 mg·kg^−1^ BW·h^−1^, 80% Met= 8.3 mg·kg^−1^ BW·h^−1^, 120% Met=14.53 mg·kg^−1^ BW·h^−1^, 200% Met=20.77 mg·kg^−1^ BW·h^−1^
